# 8‐week cognitive training intervention leads to improved mnemonic discrimination performance and near transfer from objects to scenes stimuli

**DOI:** 10.1002/alz.095262

**Published:** 2025-01-09

**Authors:** Panagiotis Iliopoulos, Boyan Rong, Eóin N. Molloy, Radoslaw Martin Cichy, Anne Maass, Emrah Düzel

**Affiliations:** ^1^ Institute of Cognitive Neurology and Dementia Research (IKND), Otto von Guericke University, Magdeburg Germany; ^2^ German Center for Neurodegenerative Diseases (DZNE), Magdeburg Germany; ^3^ Department of Education and Psychology Freie Universität Berlin, Berlin Germany; ^4^ Otto‐von‐Guericke University, Magdeburg Germany; ^5^ Division of Nuclear Medicine, Department of Radiology & Nuclear Medicine, Faculty of Medicine, Otto von Guericke University, Magdeburg Germany; ^6^ Center for Behavioral Brain Sciences (CBBS), Magdeburg Germany; ^7^ Otto von Guericke University, Magdeburg Germany; ^8^ Institute of Cognitive Neuroscience, University College London (UCL), London United Kingdom

## Abstract

**Background:**

Episodic memory declines in old age. Successful memory relies on the process of mnemonic discrimination (MD) to establish distinct representations. However, the scope for improvement in older adults’ cognitive performance using cognitive training is poorly understood. Here we investigated whether cognitive training leads to improvement in MD and whether the benefits transfer across different stimulus types, tasks and cognitive functions

**Method:**

112 older adults (age M = 67.96, SD = 3.86) completed an 8‐week web‐based MD training intervention divided into 3 groups: one group training with object stimuli (OG), one group training with scene stimuli (SG), one active control group. The training was based on the object‐scene MD task which involves differentiating similar objects and scenes (‘lures’; correct response: ‘new’) from repeated items ('repeats’, correct response: ‘old’). Stimuli were first presented in a 2‐back set‐size: the first two stimuli were new images, while each of the subsequent two could be either a lure or a repeat trial. Then set‐size increased progressively across the training. Pre‐ and post‐training, the subjects performed a behavioral session including the 2‐back object‐scene MD task and several other transfer tasks. The latter included the mnemonic similarity task (MST; i.e. a different MD task), a pattern completion, working memory, spatial learning, and navigation task.

**Result:**

Our results show a training‐induced improvement in the object‐scene MD task performance but not the MST. We found a bias‐corrected performance improvement (d prime) driven by the correct lures. Compared to the control group, the OG improved their MD performance in objects while the SG improved in scenes, but only the OG showed near transfer improvement to the non‐trained category (i.e. scenes). We found no significant training effect in other transfer tasks.

**Conclusion:**

An 8‐week cognitive training intervention led to MD improvement in the trained task. We found a same modality improvement with the OG improving in objects and the SG in scenes, and evidence of near transfer from object to scenes but not vice versa. Cognitive training can lead to MD improvement in older adults. Longer‐duration interventions are needed to assess far‐transfer effects.

